# Yellow River water rebalanced by human regulation

**DOI:** 10.1038/s41598-019-46063-5

**Published:** 2019-07-04

**Authors:** Yaping Wang, Wenwu Zhao, Shuai Wang, Xiaoming Feng, Yanxu Liu

**Affiliations:** 1 State Key Laboratory of Earth Surface Processes and Resource Ecology, Faculty of Geographical Science, Beijing Normal University, Beijing, 100875 P.R. China; 2 Institute of Land Surface System and Sustainability, Faculty of Geographical Science, Beijing Normal University, Beijing, 100875 P.R. China; 3 State Key Laboratory of Urban and Regional Ecology, Research Center for Eco-Environmental Sciences, Chinese Academy of Sciences, Beijing, 100085 P.R. China

**Keywords:** Hydrology, Environmental social sciences, Sustainability

## Abstract

The streamflow of major global rivers changes under the influences of climate change and human activities and varies greatly in different regions. The Yellow River has undergone a dramatic shift during the last six decades. Its streamflow gradually dwindled away and even dried-up severely in the late 20th century, but in recent years it has recovered and remains stable. Comprehensive understanding of the river streamflow change and its driving forces promotes effective water resource management within this complex human-natural system. Here, we develop a runoff identity attribution approach to analyze 61 years of streamflow observations from the Yellow River. We find that between the 1950s and the 1980s, human water consumption contributed more than 90% to streamflow reduction, but from the 1970s onwards, land cover change became the major factor to decrease streamflow. Since 2000, government management schemes have prevented streamflow from declining further and guarantee its stability. Based on the analysis framework we propose, persistent droughts, which are related to abrupt streamflow abatement, may be the most uncontrollable factor in the future. A more resilient management system should be therefore built to grapple with the expected increased frequency of such extreme climate events in the future.

## Introduction

Streamflow is a major source of water for human consumption, and also the most concise indicator of a basin’s response to climate change and human activities^1,2^. Most continental streamflow generally increased during the 20^th^ century, as the global hydrological cycle intensified, driven by atmospheric variation and land use change^3–5^, but there is uncertainty regarding streamflow trends on the regional scale^6,7^. At these scales, spatial heterogeneity and social-ecological interactions make the process of streamflow change more complex^8,9^. In general, precipitation and its intensity relating to large-scale atmospheric circulation, and temperature are the major climatic factors causing streamflow change^10–16^, while physical features of landscape and human activities such as urbanization, agricultural development, and water-soil conservation measures also play important roles in altering streamflow^17–23^. Although there are many researches focusing on regional streamflow change drivers, previous studies pay more attention to one or two aspects. Therefore, holistic analysis of regional streamflow features and mechanisms is required to promote a better understanding of the basin-scale water budget, which is critical for sustainable water resource management^24^.

**Figure 1:**
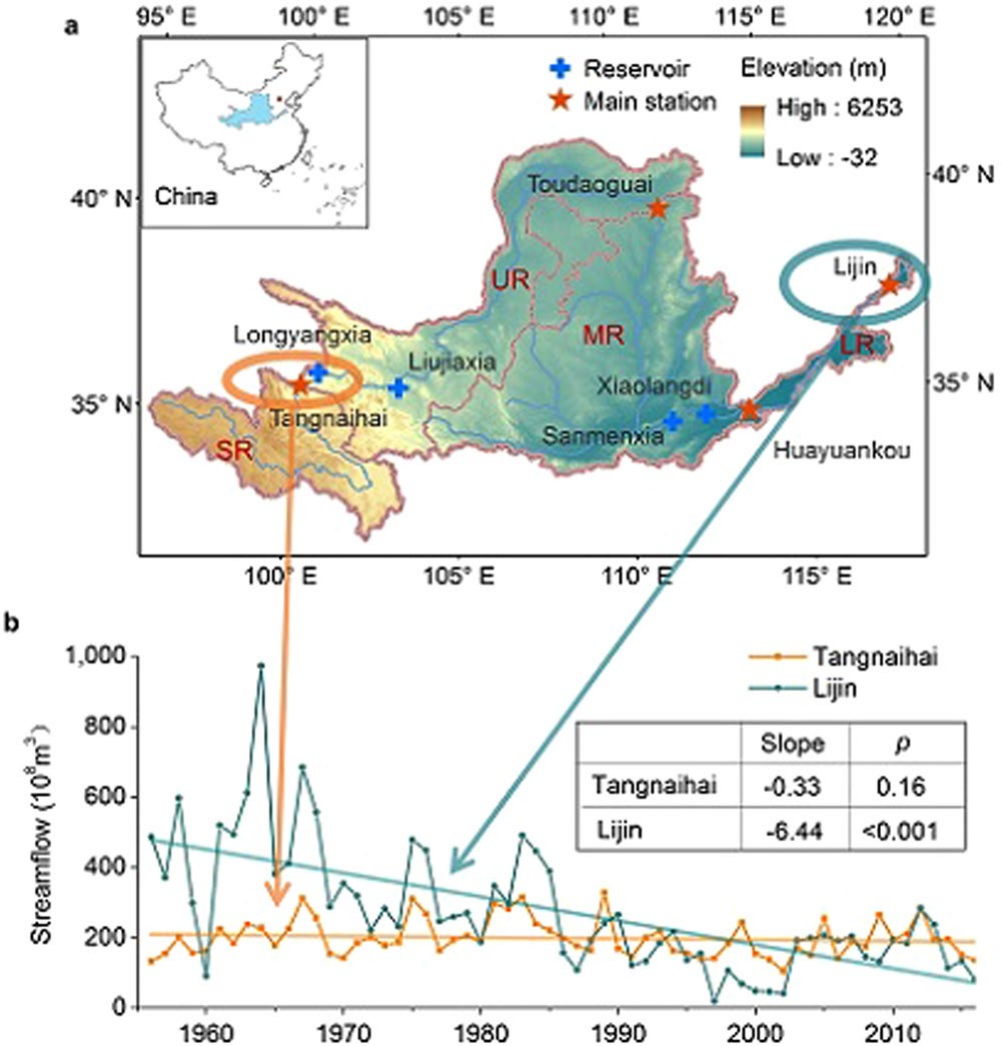
Location of the Yellow River (YR) basin and annual streamflow of the YR in the past six decades. (**a**) The pentagrams represent the control gauging stations of the source regions (SR), upper reaches (UR), middle reaches (MR) and lower reaches (LR), and the crosses represent the main reservoirs on the YR. (**b**) The time series of annual streamflow at Tangnaihai station and Lijin station, the unit of slope is 10^8^ m^3^. New (prepared by YW in ArcMap 10.2, https://www.esri.com/zh-cn/arcgis/products/arcgis-pro/resources).

The Yellow River (YR) (Fig. 1a), flowing through the arid and semi-arid areas of northern China and sustaining a population of 114 million people^25^, faces an enormous challenge of water shortage, especially in the period of great changes in social-ecological environment^26,27^. With the increase of population and development of social economy, there has been significant growth in water consumption, including agricultural, industrial, and domestic water use, along with water facilities during the last six decades^28,29^. Serious soil erosion in the Loess Plateau, mainly located in the middle reaches of the YR basin, and high sediment load in the YR have been a major issue for the local population for many years^26,30,31^, the Chinese Government implements a series of large ecological engineering and invests more than US$8.7 billion^32^ to solve this problem. After a long-term water-soil loss control, the Loess Plateau has become greener and the sediment load of the YR has significantly decreased in recent decades, but this decrease is associated with streamflow reduction^31–36^. Climate factors, such as increasing temperature, decreasing precipitation and changing extreme climate events, also aggravate water scarcity in the YR^37–39^. The streamflow has decreased severely; the lower YR even dried-up completely in the late 20^th^ century^40,41^.

Lots of studies have taken notice of this phenomenon and there is an agreement on the significant roles of precipitation, human abstraction and water-soil conservation measures such as afforestation in streamflow decrease^28,29,42–45^. However, with great efforts under the government management, water use regime of the Yellow River (YR) was altered after acute dried-up events, and the declining streamflow trend has been reversed immediately^26,46–49^. In spite of these major changes, there have been few systematic studies focusing on long-term streamflow change in the YR basin and its response to various driving factors in different periods.

The objective of this study is therefore to understand the temporal change and spatial distribution of streamflow in the Yellow River basin, and to quantify the influences of precipitation, potential evapotranspiration, land cover and direct human activities on streamflow change during the last six decades.

## Results

### General observations and water budget

Streamflow in the Yellow River (YR) estuary has significantly decreased (slope −0.83 mm·yr^−2^, *p* < 0.001) since 1956, and, in recent years, there has been no significant difference (*p* = 0.28) between the streamflow of the YR estuary and the source regions (Fig. 1b). At the Tangnaihai gauging station (TNH) at the outlet of the YR source regions, annual streamflow has shown an insignificant change trend (slope −0.33 × 10^8^ m^3^ yr^−2^, *p* = 0.16) since 1956, while at the Lijin gauging station (LJ), the control station of the YR estuary, the streamflow fell from 501.15 × 10^8^ m^3^ yr^−1^ in the 1960s to 140.75 × 10^8^ m^3^ yr^−1^ in the 1990s, and finally increased to 175.20 × 10^8^ m^3^ yr^−1^ in the 2010s. This latter value is approximately equal to the value of 195.94 × 10^8^ m^3^ yr^−1^ measured at TNH during the 2010s, even though there are 51 major tributaries, each with a catchment area greater than 1,000 km^2^, flowing into the YR between TNH and LJ.

**Figure 2:**
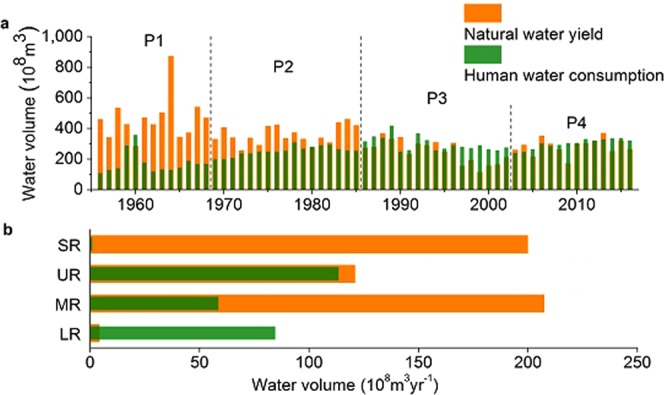
The temporal and spatial distribution of natural water yield and human water consumption in the Yellow River (YR) basin. (**a**) The time series of annual natural water yield and human water consumption. The time series has been divided into four periods according to regional streamflow features. (**b**) Average annual natural water yield and human water consumption in the source regions (SR), upper reaches (UR), middle reaches (MR) and lower reaches (LR) in the past six decades.

Analysis of the water budget of the region between Tangnaihai gauging station (TNH) and Lijin gauging station (LJ) (see Methods) revealed that its regional streamflow had a significant decline (slope −6.07 × 10^8^ m^3^ yr^−2^, *p* < 0.001) during the period 1956–2016. This decline was the result of both increasing human water consumption (slope 2.80 × 10^8^ m^3^ yr^−2^, *p* < 0.001), and decreasing natural water yield (slope −3.37 × 10^8^ m^3^ yr^−2^, *p* < 0.001). Based on 7-year moving-average annual regional streamflow between TNH and LJ, we divided the past 61 years into four periods covering 1956–1968 (P1), 1969–1985 (P2), 1986–2002 (P3), and 2003–2016 (P4) (Fig. 2a; Supplementary Fig. 1). In this region, natural water yield exceeded human water consumption greatly (*p* < 0.001) during P1. Their gap narrowed but was still significant (*p* < 0.001) in P2, but disappeared, and even reversed for P3 and P4, because water consumption was close to or above water yield between these two stations.

In summary, regional streamflow at the two stations roughly balanced twice during two periods in P3 and P4. This balance was first achieved (see Methods, *p* = 0.45) during 1986–1996 in P3, because decreasing natural water yield equalled to increasing human consumption. As these trends continued, the balance was lost in 1997, but then recovered (*p* = 0.28) in P4 owing to a halt to the trends and a slight increase of natural water yield. An interesting difference between P3 and P4 was that the drying-up of the lower YR occurred frequently and for increasing number of days in the former period, but no drying-up occurred in the latter period at all (Supplementary Fig. 2).

Spatial variations between natural water yield and human water consumption exist in the Yellow River (YR) basin (Fig. 2b). During 1956–2016, the YR had an average natural water yield volume of 532.82 × 10^8^ m^3^ yr^−1^, with 38.94% coming from the middle reaches (MR), 37.54% being attributed to the source regions (SR), 22.72% to the upper reaches (UR), and only 0.80% to the lower reaches (LR). This difference comes from the various physical features of these sub-regions (Supplementary Table 1). The SR, UR and MR all have large catchment areas with a great quantity of water flowing into the river, ratios of their areas to the whole basin are 15.23%, 33.53% and 42.34% respectively. Although the UR is larger than SR in area, it flows through the arid zone of Ulan Buh Desert and Kubuqi Desert where precipitation is low but evaporation is high. As for the LR, its small area is due to uplifted riverbed resulting from heavy sediment of the Loess Plateau. This region has been the watershed between Hai River basin and Huai River basin, and the lower YR has been a suspended river with little catchment.

However, spatial distribution of human water consumption doesn’t match water yield pattern well. Locating in arid and semi-arid regions mostly, agricultural water in the Yellow River (YR) basin accounts for more than 70% of the total human water consumption. Irrigation is the most important mode of agricultural development, especially in upper reaches (UR). Besides farmland within the basin, the lower YR also provides water for irrigation areas in the North China Plain (Supplementary Table 1). Accordingly, human water consumption of the source regions (SR), upper reaches (UR), middle reaches (MR) and lower reaches (LR) contributed 0.38%, 44.04%, 22.76% and 32.82% respectively, to the total amount of water consumption, 257.81 × 10^8^ m^3^ yr^−1^, in the whole basin. This mismatch between water yield and consumption is particularly serious in SR and LR, with the available water supply in LR being almost wholly reliant on flow from upstream (95%).

### Runoff identity analysis

**Figure 3:**
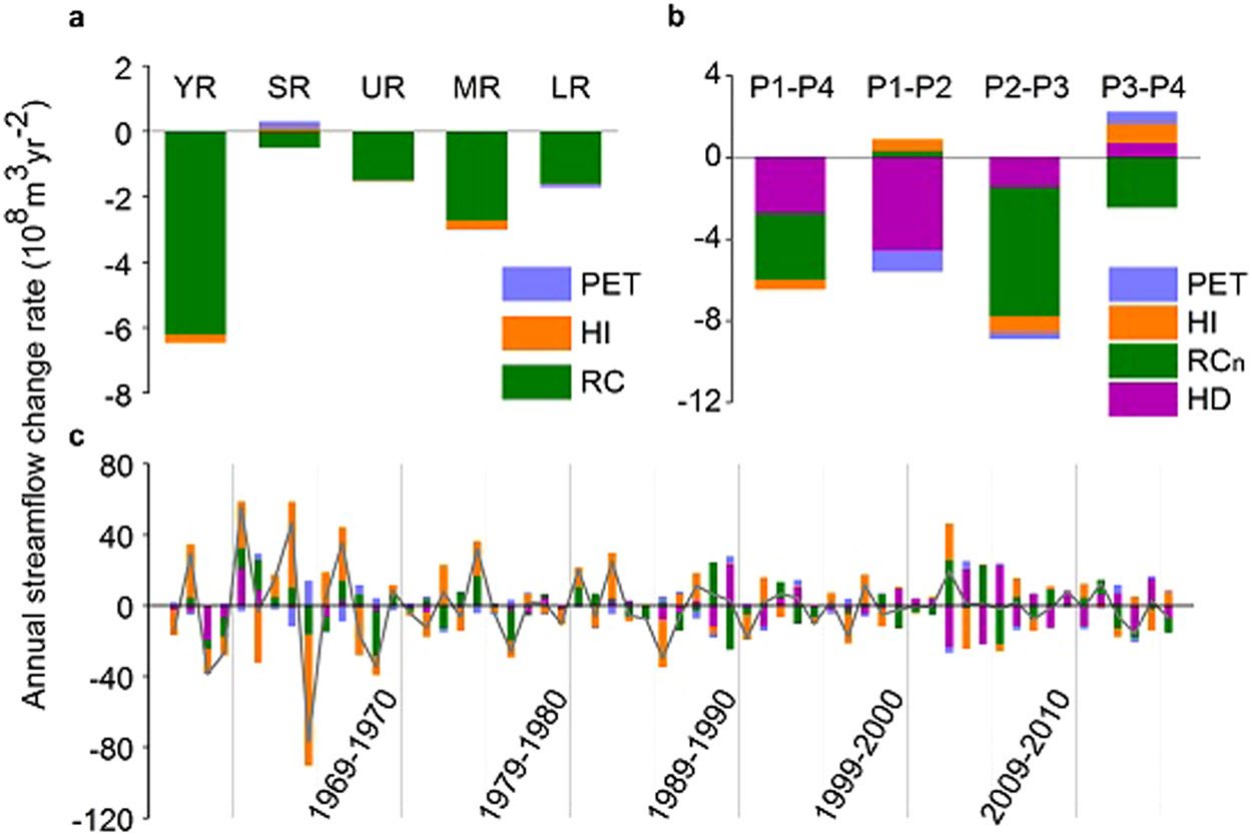
Contributions of runoff identity factors to annual streamflow change rates. (**a**) Partial annual streamflow change rates due to potential evapotranspiration (PET), hydrothermal index (HI), and runoff coefficient (RC) in the Yellow River (YR), source regions (SR), upper reaches (UR), middle reaches (MR) and lower reaches (LR). (**b**) Partial annual streamflow change rates due to PET, HI, natural runoff coefficient (RC_n_) and direct human disturbance (HD) from P1 to P4 (1956–2016), P1 to P2 (1956–1985), P2 to P3 (1969–2002), and P3 to P4 (1986–2016). **c**, Partial annual streamflow change rates due to PET, HI, RC_n_ and HD between two consecutive years during 1956 and 2016.

We attributed regional streamflow change to changes of regional potential evapotranspiration (PET), hydrothermal index (HI), and runoff coefficient (RC) based on runoff identity (see Methods; Fig. 3a; Supplementary Fig. 3 and Supplementary Table 2). The proportional change rate of streamflow in the Yellow River (YR) basin was −2.34% yr^−1^, with 96.46% of this change resulting from decreasing RC, and only 3.54% being due to changes in HI and PET. The middle reaches (MR) made the largest contribution to the streamflow decline of the YR (46.75%), with a proportional change rate of −2.02% yr^−1^. RC accounted for 90.34% of this change, while HI made up 9.23% and PET only 0.43%. Together the lower reaches (LR) and upper reaches (UR) contributed to more than half of the YR streamflow reduction (26.60% and 23.45%, respectively) and in both cases decreasing RC was again the main driver. The remaining 3.20% of the reduction in the YR was attributed to the source regions (SR) where streamflow dropped at a proportional rate of only −0.10% yr^−1^. The proportional rate of decreasing RC in the SR was −0.24% yr^−1^, more than twice that of the streamflow, while PET and HI both increased, with proportional rates of 0.09% yr^−1^ and 0.05% yr^−1^ respectively.

These results highlighted the dominant role of runoff coefficient (RC) in controlling the Yellow River (YR) streamflow reduction. To gain further insight, we divided the effect of RC into two parts, natural runoff coefficient (RC_n_) and direct human disturbance (HD), and then specified the major contributing factors among different periods (see Methods; Fig. 3b; Supplementary Fig. 4 and Supplementary Table 3). During P1 and P4, RC_n_ accounted for 50.45% of the streamflow decrease in the YR, with HD contributing 42.63%, and the remaining 6.92% being attributable to potential evapotranspiration (PET) and hydrothermal index (HI). For P1 and P2, the rate of decline of the streamflow was −0.61 mm·yr^−2^, in which HD made the largest contribution (96.80%). Changes in PET and HI contributed 9.53% to this decline, with the 6.33% reduction surplus being offset by increasing RC_n_. The largest decline in YR streamflow, −1.15 mm·yr^−2^, occurred between P2 and P3, and was partitioned as follows: RC_n_ (70.88%), HD (16.41%), HI (9.27%) and PET (3.44%). For P3 and P4, there was little reduction in streamflow, in comparison to the earlier periods, with a rate of only −0.03 mm·yr^−2^. In this case, RC_n_ was the only contributory factor that reduced streamflow, with the contributions of other factors all mitigating the reduction.

On the annual scale, both hydrothermal index (HI) and runoff coefficient (RC) had strong effects on streamflow variation in the Yellow River (Fig. 3c; Supplementary Table 4). The long-term trend of streamflow change masked short term fluctuations, so we explored the contributions of the runoff identity factors to streamflow difference between consecutive years. On average, for the 60 pairs of consecutive years considered, the changes in hydrothermal index (HI), natural runoff coefficient (RC_n_), direct human disturbance (HD) and potential evapotranspiration (PET) accounted for 40.38%, 28.79%, 23.70% and 7.13% respectively, of the inter-annual streamflow variation. A significant reduction over time was found for the contributions of HI and PET (*p* < 0.001 and *p* < 0.05, respectively), but the HD and RC_n_ contributions showed an increasing trend (*p* < 0.001 and *p* = 0.12, respectively).

## Discussion

More than 90% of the long-term streamflow decline of the Yellow River (YR) over the last six decades was attributed to runoff coefficient (RC) reduction. The dominant processes behind this reduction have changed from direct human disturbance (HD) to natural runoff coefficient (RC_n_). Human water consumption is the major component of HD, it reduced streamflow significantly (*p* < 0.001; Supplementary Fig. 5), especially during P1 and P2, when it increased at a rate of 4.57 × 10^8^ m^3^ yr^−2^ almost balancing the streamflow decrease rate of −4.72 × 10^8^ m^3^ yr^−2^ at the same stage. This increase in consumption was associated with the increase of irrigation areas in the YR basin from only 8,000 km^2^ in the 1950s to 63,000 km^2^ in the 1980s^25^. However, from P3 to P4, human water consumption was relatively stable (slope 1.35 × 10^8^ m^3^ yr^−2^, *p* = 0.93; Supplementary Fig. 5), taking up about 66% of the natural streamflow of the YR but contributing little to its temporal change. Another measure of direct human disturbance (HD) affecting streamflow is reservoir regulation, which contributes more to the change of seasonal distribution of streamflow, rather than its long-term volume variation.

During P2 and P4, declining natural runoff coefficient (RC_n_) related to dramatic land cover change taking place in the Yellow River (YR) basin, largely accounted for the reduction in streamflow. The YR once carried more fluvial sediment than any other rivers in the world, which led to the launch of large-scale ecological projects in the YR basin, such as the Conservation of Water and Soil Ecological Engineering (CWSEE) project and the Grain-for-Green Programme (GGP), to prevent soil erosion^50,51^. After the implementation of GGP in 1999, difference of the normalized differential vegetation index (NDVI) has increased in more than 61% the YR basin (Supplementary Fig. 6). Official statistics show that the ratio of the cumulative area of soil and water conservation in the YR basin has increased from 3.06% in 1969 to 51.36% in 2012 (Supplementary Fig. 7). Terraces, check dams, small reservoirs and afforestation are major measures used in these projects^31,52^. Wang *et al*.^53^ calculated that, on average, 5.01 × 10^8^ m^3^ yr^−1^ of water was retained by such measures before 1997, and their water-holding capacity experienced rapid growth in P2. Based on a method proposed by Liu *et al*.^54^, we estimated that the augmentation of afforestation caused streamflow decline from a rate of −2.16 × 10^8^ m^3^ yr^−2^ in P2 and P3 to −2.54 × 10^8^ m^3^ yr^−2^ in P3 and P4. Satellite-based land use images of the YR basin also shown that farmland, construction land and woodland changed a lot between 1980 and 2015, while the change of farmland and woodland always took place in the Loess Plateau where soil erosion was severest (Supplementary Fig. 8).

**Figure 4:**
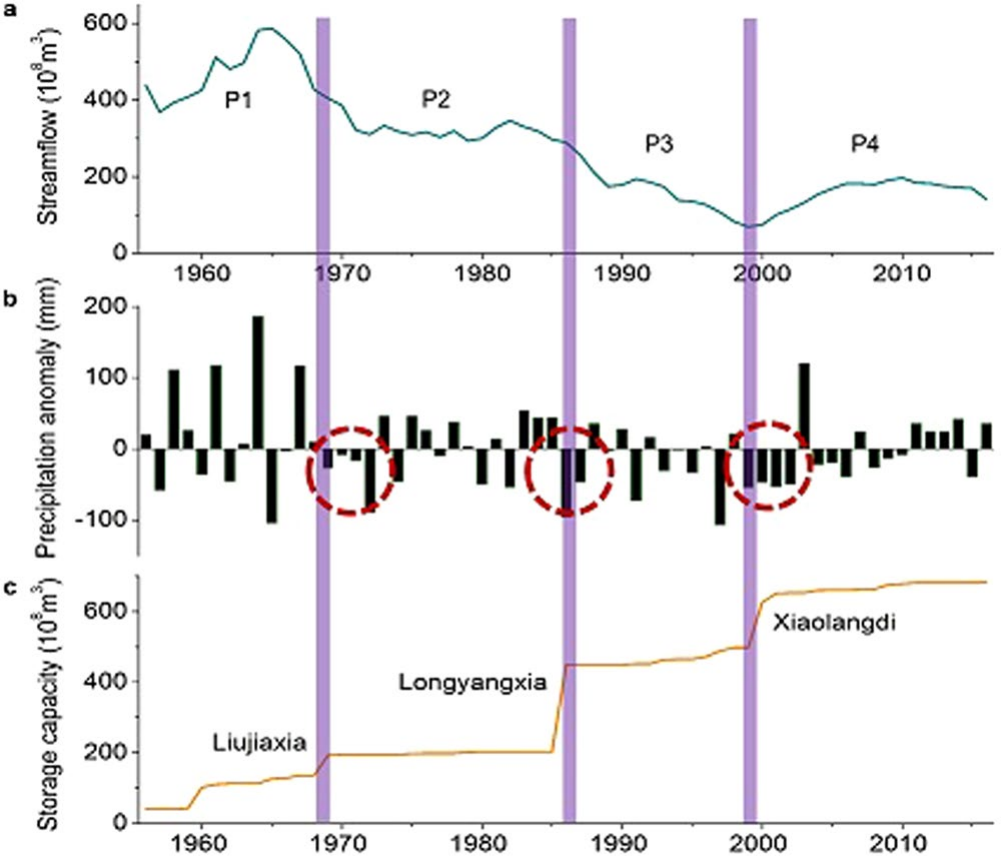
Abrupt streamflow decline relating to persistent drought and large reservoir construction. (**a**) 7-year moving-average annual streamflow at the Yellow River (YR) estuary. (**b**) Precipitation anomalies in the YR basin. The red circles represent persistent drought events. (**c**) Cumulative storage capacity of the YR basin. Purple lines show that the years of sharp streamflow reduction are coincident with persistent drought events and the first impounding times of Liujiaxia, Longyangxia and Xiaolangdi reservoirs.

In addition to the long-term streamflow decrease described above, abrupt streamflow reduction occurred at the transitions between periods of water budget (Fig. 4a; Supplementary Fig. 1) and we explored the possible reasons for the differentiation between periods. The relationship between cumulative precipitation and cumulative runoff (Supplementary Fig. 9) shown four steep falls around the years 1969, 1986, 1997 and 2003. These years coincided with persistent drought events (Fig. 4b) and the first impoundment of large reservoirs (Fig. 4c) in the Yellow River (YR) basin. Persistent drought occurs when the cumulative percentage of precipitation anomalies more than one year exceeds −30 percent and it directly reduces water supply of the basin in drought years. Moreover, a recent study suggested that persistent drought events also significantly decreased runoff coefficient (RC) in following years compared to the single-year drought which just declined precipitation of the drought year^55^. So, it’s not just a coincidence that persistent drought occurred at times of water budget transition periods. Persistent drought not only led to abrupt streamflow decline, also aggravated differentiation between two periods by altering precipitation-runoff relationship. New reservoirs store large quantities of water within a short time as a prerequisite for the reservoir running and this initial phase reduces downstream flow sharply. Liujiaxia, Longyangxia and Xiaolangdi are the first three reservoirs in terms of storage capacity in the YR basin (Supplementary Table 5), they impounded lots of water at their first impounding years, 1968, 1986 and 1999 respectively, which partially contributed to abrupt reductions accordingly. Afterwards they also promoted changes in runoff coefficient (RC) by regulating and redistributing seasonal streamflow, for example by storing floodwater which then became an available water resource for irrigation in growing season^56–59^. As a result, the ratio of flood-season flow volume to annual streamflow and the variation coefficient of flood-season flow both decreased after the building of these reservoirs (Supplementary Fig. 10). Although it’s hard to quantify the effects of persistent drought and reservoir construction on the streamflow decline in subsequent periods, they should never be ignored in YR water budget changes of different periods.

In P4 the water balance was recreated; streamflow reduction was suspended and drying-up episodes no longer occurred (Fig. 1 and Supplementary Fig. 2). Government management was the critical factor in achieving this turnaround: in order to sustain stable and continuous streamflow, human water use regime was adjusted, in terms of abstraction and storage, to offset the influences of climate factors and natural runoff coefficient (RC_n_). During the late 20^th^ century, human water consumption had reached the maximum level that the Yellow River (YR) could bear^60^, and drying-up episodes in the lower YR were becoming more frequent and of longer duration (Supplementary Figs 2 and 5). The situation came to a head with the severe drought of 1997, 90% of the downstream segment (704 km) had no water for a period of 226 days. To prevent the situation from deteriorating further, in 1998 the Chinese Government promulgated and implemented an annual allocation and regulation scheme for available water volume in the Main Yellow River. The scheme specified the maximum water intake volume allowed for each province along the YR, and authorized the Yellow River Conservancy Commission (YRCC) to operate the scheme holistically based on climatic conditions^61^. Under the scheme, the YRCC gives priority to ensuring that sufficient ecological water is retained in the river and then allocates the remaining part of the available water to various provinces along the YR. With the completion of the Xiaolangdi hydropower station in 2001, the Longyangxia-Liujiaxia-Sanmenxia-Xiaolangdi reservoirs system of the YR basin was brought into operation. The total storage capacity of the YR basin increased from 40 × 10^8^ m^3^ before 1956 to 682 × 10^8^ m^3^ in 2016 (Fig. 4c). This system improves government capacity for water regulation and provides technical support for the implementation of the Water Diversion and Sediment Regulation Project (WDSRP) which aims to scour away sediments and keep the YR flowing^26,46,47^. The cooperation between all of these measures including institutions, policies and facilities has prevented the YR from drying-up and kept its streamflow stable on both inter-annual and intra-annual scales (Fig. 1 and Supplementary Fig. 10). It appeared that the occurrence and loss of water balance of the YR in P3 was inexorable due to the increasing water consumption and the intensification of the contradiction between water supply and demand. And the water balance subsequently achieved in P4 was a great achievement of government management, a series of measures have made the YR much more resilient to risks such as drying-up and flooding.

**Figure 5:**
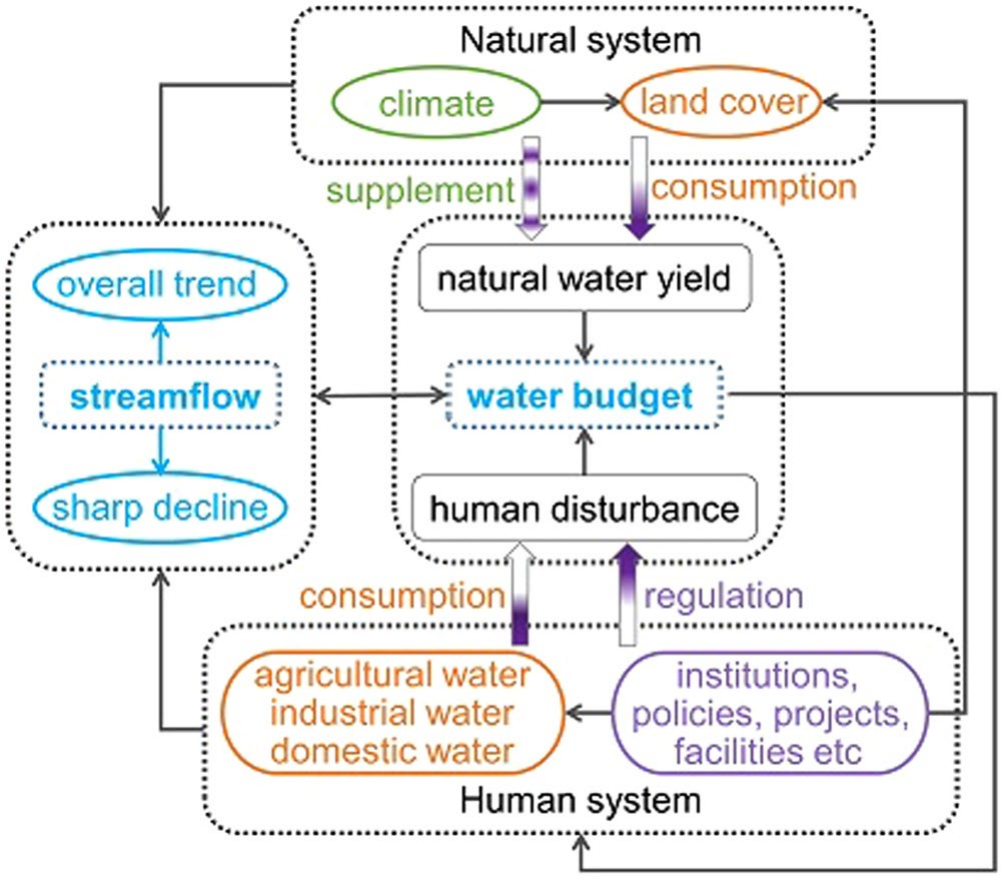
Analysis framework in this study. Water budget is determined by natural water yield and human disturbance, and it can be illustrated by regional streamflow change. The streamflow of the Yellow River has an overall decrease and several periods of sharp reduction in recent decades. The relationship between the coupled human-natural system and the water budget has been found. Note that the gradient fill of arrows represents the change of impact level, a light color means weaker effects while the darker color represents stronger effects.

In this study, we have provided a unique perspective on the Yellow River (YR) water budget and its change, and also presented a simple and efficient method for attribution analysis to capture the main streamflow characteristics in response to the key events and factors which dominate the available water resource. According to our analysis framework (Fig. 5), it is a strong possibility that the current water balance of the YR basin will remain in place for the next few years. Currently, the storage capacity of large reservoirs in the YR is 465 × 10^8^ m^3^, and there are a number of other large reservoirs, with total storage capacity of more than 313 × 10^8^ m^3^, in the planning stages. This increase in storage capacity should further strengthen the competence of government in available water management^25^. Additionally, the development and operation of water-saving technologies will mediate the contradiction between water demand and supply^62^, and the Yellow River Conservancy Commission (YRCC) will continue executing the water allocation scheme to get human water consumption under control and sustain a healthy fluvial system^25^. Net primary productivity in the Loess Plateau, a major part of the YR basin, is approaching its sustainable water resource limits^32^, and ecological projects have imposed measures to alleviate water consumption. However, in spite of the progress made in YR management in the last few years, extreme climate events caused by global change are likely to be more frequent in the future^63–65^, and abrupt streamflow changes associated with uncontrollable climate factors will be a major concern over the coming decades. The management system must be perfected and made resilient to cope with these future climate challenges.

## Methods

### Data

Yearly and monthly streamflow data from the main gauging stations along the Yellow River (YR) for the period 1956 to 2016 were obtained from the Yellow River Conservancy Commission (YRCC). Yearly human water consumption and storage capacity of the YR basin, and partial large reservoirs water storage data for the 1956 to 2016 period were collected from papers^28,29,66–68^ and from the Yellow River Water Resources Bulletin (http://www.yellowriver.gov.cn/other/hhgb/). Daily meteorological data from 1956 to 2016, including precipitation, maximum and minimum temperatures, wind speed, atmospheric pressure, relative humidity and duration of sunshine, were provided by the China Meteorological Data Service Center (http://data.cma.cn/). Data from 319 meteorological stations were involved in this research. Daily potential evapotranspiration at each station was estimated with the Penman-Monteith equation, and yearly potential evapotranspiration and precipitation were accumulated day by day. The Kriging interpolation was used to calculate regional average precipitation and potential evapotranspiration. Records of soil and water conservation measures, including areas of terraces, dams and vegetation, were collected from the Yearbook of the Yellow River (1990–2012), the China Water Conservancy Yearbook and other publications^53^. Satellite-based land use images (1980, 2015) and normalized differential vegetation index images (1998, 2015) of the YR basin were downloaded from Resource and Environmental Data Cloud Platform (http://www.resdc.cn/)^69,70^.

### Regional water budget

To depict the water budget, we used regional streamflow that was calculated by subtracting the gauged streamflow at the entrance to a region from that at the outlet (regional streamflow = streamflow_outlet_ − streamflow_entrance_). Human water consumption and reservoir water storage change were then added to the regional streamflow to get the regional natural water yield (natural water yield = regional streamflow + human water consumption + reservoir water storage change). Natural water yield represents the initial streamflow of a region before human abstraction and regulation through water facilities.

In this paper, we divided the Yellow River (YR) basin into four regions based on the topographic features and human activity; the source regions (SR); upper reaches (UR); middle reaches (MR); and lower reaches (LR). They cover areas of 12.40 × 10^4^ km^2^, 27.30 × 10^4^ km^2^, 34.47 × 10^4^ km^2^ and 3.00 × 10^4^ km^2^, respectively, that account for 15.23%, 33.53%, 42.34% and 3.68%, respectively, of the total area of the YR basin. The remaining 5.22% of the area belongs to the interior drainage basin of the Ordos Plateau which is excluded from this article. The annual precipitation of the YR basin ranges from 200 mm to 650 mm, and decreases from the southeast to northwest. So the UR is the driest region in the YR basin, and the upper YR flows through the Hetao Plain that has large irrigation areas, as well as Ulan Buh Desert and Kubuqi Desert. As a part of the Tibetan Plateau, the SR is the major water resource of the YR basin with sparse population, and it’s sensitive to climate change owing to special geographical features. The MR mainly lies to semi-arid areas and faces serious soil erosion, it’s also the traditional cultivated region of China with a dense crowd. Because of uplifted riverbed, the LR covers a small but flood-prone areas, the lower YR provides water for large irrigation areas in the North China Plain (Supplementary Table 1).

Tangnaihai gauging station (TNH), Toudaoguai gauging station (TDG), Huayuankou gauging station (HYK) and Lijin gauging station (LJ) are the outlets of SR, UR, MR and LR respectively, while TNH, TDG and HYK are also the entrances to UR, MR and LR respectively (Fig. 1a). We calculated the regional streamflow and natural water yield of each region by using the data from these gauging stations (Fig. 2).

### Runoff identity factors decomposition

Analogous to the Kaya and Sediment Identity^31,71,72^, the runoff (R) can be considered as the product of the three variables:

1 $$R=PET(\frac{P}{PET})(\frac{R}{P})=PET\cdot HI\cdot RC,$$

where R, PET, P, HI and RC are abbreviations for runoff, potential evapotranspiration, precipitation, hydrothermal index and runoff coefficient, respectively. So runoff identity has been constructed.

We then defined the proportional change rate of a variable X(t) as r(X) = (dX/dt)/X. Accordingly, r(R) = ((dR/dt)/R) = ((d(PET·HI·RC)/dt)/(PET·HI·RC)) = ((dPET/dt)/PET) + ((dHI/dt)/HI) + ((dRC/dt)/RC), the counterpart of the runoff identity for proportional change rates can be rewritten as

2 $$r(R)=r(PET)+r(HI)+r(RC).$$

Therefore, we attributed regional runoff change to the changes of potential evapotranspiration (PET, denoting atmospheric water demand), hydrothermal index (HI, the ratio of precipitation to potential evapotranspiration, denoting natural water supply), and runoff coefficient (RC, the ratio of runoff to precipitation, denoting water yield ability) by using this attribution approach.

With long time series of R, PET, HI and RC observations, we calculated their proportional change rates in different periods using an adjusted Sen’s slope method^73^:

3 $$r(X)=\frac{Mean\frac{{x}_{j}-{x}_{i}}{j-i},\,\forall j > i}{\bar{X}},$$

where *x*_*j*_ and *x*_*i*_ are the j^th^ and i^th^ of *X* observations respectively and $\bar{X}$ is the average of the series of *X* observations. The theoretical proportional change rate, the sum of the proportional change rates of PET, HI and RC, closely approximates the actually calculated proportional change rate of R (Supplementary Fig. 11). The relative contribution of each identity factor is the ratio of its proportional change rate to the theoretical proportional change rate of R during the same period. We then multiplied the contribution ratio by the change rate of runoff to get the partial change rate of runoff related to each factor.

Streamflow (m^3^) is proportional to runoff (mm) and their trends are essentially identical, so the relative contribution of each identity factor to the change rate of runoff is equivalent to its contribution to streamflow. Here we focused on the influence of each identity factor on streamflow.

Identity attribution approaches have been successfully used in many fields, such as Forest Identity for carbon sequestering in forests^74^ and Sediment Identity for reduced sediment transport in the Yellow River^31^, due to their simple mathematical forms and great capabilities of decomposing factors without residuals^75^. However, these kind of methods only focus on the contribution of each factor to the change rates of research variables rather than their quantity and these factors are interdependent, which means the method is more sensitive to changeable factors and their contributions should be further explained with intrinsic mechanism based on specific drivers or processes. As for runoff identity, it is therefore suitable for regions where water resources have experienced a dramatic change and it’s better if the change is monotonic, like the increasing carbon emissions. However, hydrometeorologic factors fluctuate widely and reasonable phase division is important for attribution analysis to capture the critical drivers affecting the hydrological change in different stages.

### Human disturbance decomposition

Runoff coefficient (RC), namely the ratio of runoff to rainfall, used to describe the water yield ability of a region, can be altered by natural processes (RC_n_) such as vegetation interception caused by land cover change, and direct human disturbance (HD) including water abstraction and regulation. To separate HD from RC, we reconstructed the natural streamflow through yearly gauged data (natural streamflow = gauged streamflow + human water consumption + reservoir water storage change) and calculated its change rate. The difference in change rates between gauged and natural streamflow was attributed to HD, and the remaining part of the partial change rate of streamflow caused by RC was interpreted as the influence of RC_n_.

### Statistical analysis

The Mann-Kendall test was used to analyze the change trends of yearly streamflow, precipitation, potential evapotranspiration and other hydrometeorological data^76,77^, while Sen’s slope method was used to calculate their change rates^73^. We applied a paired t-test to evaluate the streamflow difference between two gauging stations, and conducted single-sample t-tests to determine whether regional streamflow change is significantly different from 0 (if its water budget balances). A nonparametric test, the Kruskal-Wallis test, was used to test the differences between the four study periods.

## Supplementary information

Yellow River water rebalanced by human regulation

## Data Availability

The data analyzed during the current study are available in the Yellow River Conservancy Commission (http://www.yellowriver.gov.cn) and the China Meteorological Data Service Center (http://data.cma.cn/).
