# Supplementary material for: Yellow River water rebalanced by human regulation
Source: Sci Rep. 2019 Jul 4;9:9707. doi: 10.1038/s41598-019-46063-5 (PMC6609694; doi:10.1038/s41598-019-46063-5)
Supplement: Supplementary file 1 — Yellow River water rebalanced by human regulation [file 41598_2019_46063_MOESM1_ESM.pdf]

Yellow River water rebalanced by human regulation

Yaping Wang[1,2], Wenwu Zhao[1,2], Shuai Wang[1,2*], Xiaoming Feng[3], Yanxu Liu[1,2]

[1] State Key Laboratory of Earth Surface Processes and Resource Ecology, Faculty of Geographical Science, Beijing Normal University, Beijing 100875, P.R. China

[2] Institute of Land Surface System and Sustainability, Faculty of Geographical Science, Beijing Normal University, Beijing 100875, P.R. China

[3] State Key Laboratory of Urban and Regional Ecology, Research Center for Eco-Environmental Sciences, Chinese Academy of Sciences, Beijing 100085, P.R. China

Supplementary Information

Supplementary Fig. 1
Supplementary Fig. 2
Supplementary Fig. 3
Supplementary Fig. 4
Supplementary Fig. 5
Supplementary Fig. 6
Supplementary Fig. 7
Supplementary Fig. 8
Supplementary Fig. 9
Supplementary Fig. 10
Supplementary Fig. 11
Supplementary Table 1
Supplementary Table 2
Supplementary Table 3
Supplementary Table 4
Supplementary Table 5

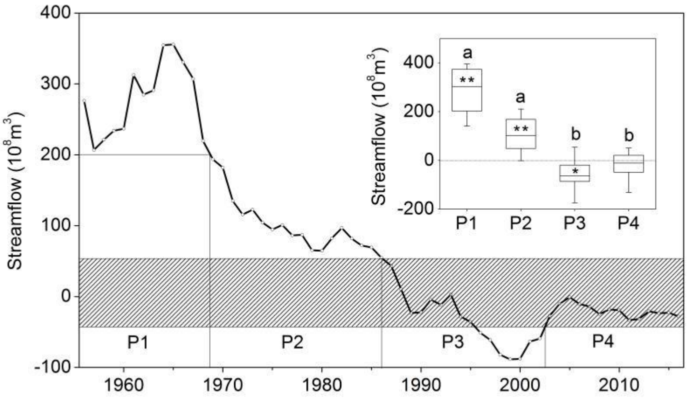

Supplementary Fig. 1 | The classification principle of the water budget between Tangnaihai station and Lijin station. 7-year moving-average annual regional streamflow is greater than 200×10^8 m^3 in P1, less than 200×10^8 m^3 and greater than 50×10^8 m^3 in P2, less than 50×10^8 m^3 in P3 and P4, but greater than -50×10^8 m^3 in P4. The shaded block represents a possibly balanced water budget. **Inset:** Comparison of annual regional streamflow over different periods. In the box-whisker plots, the central mark indicates the median, and the bottom and top edges of the box indicate the 25th and 75th percentiles, respectively. The maximum and minimum whisker lengths are specified as 1.5 times the interquartile range. A single-sample t-test was conducted to compare data of each period with 0, and the symbols * and ** indicate that the null hypothesis could be rejected at a significance level of 0.05 and 0.001. Different letters mean significant differences between different periods ($p < 0.05$, Kruskal-Wallis test).

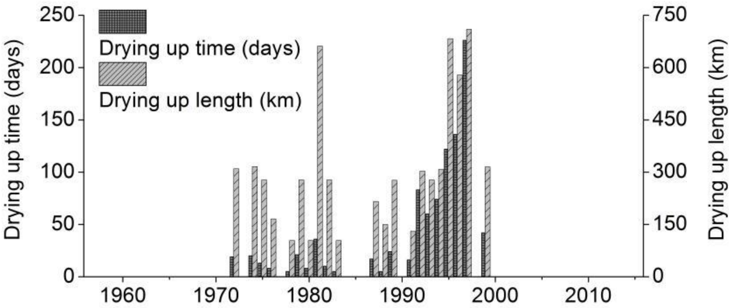

Supplementary Fig. 2 | Drying-up time and length for each year in the Yellow River in recent decades.

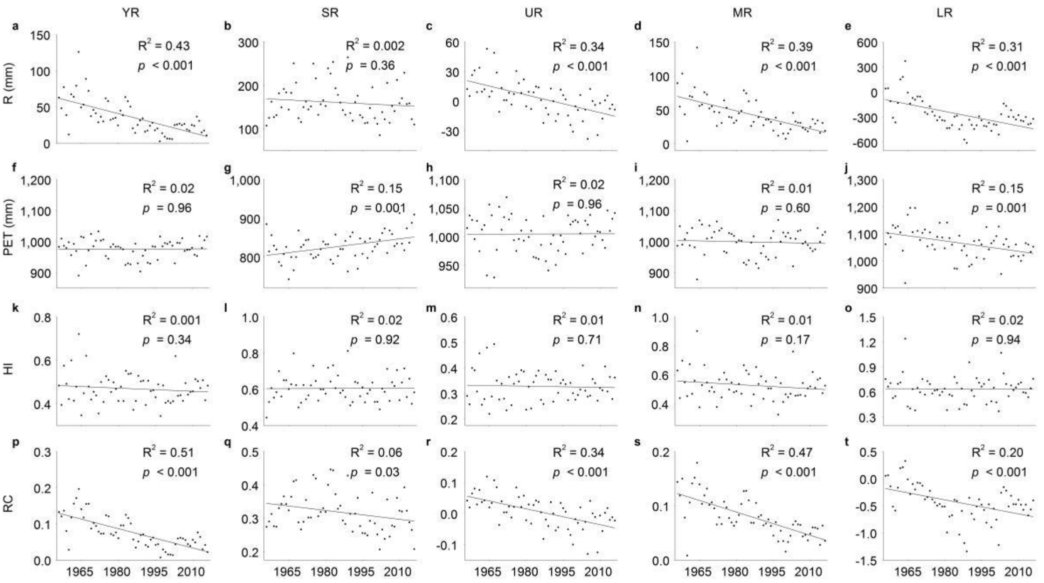

Supplementary Fig. 3 | Values of runoff and its identity factors in the last six decades. a-e, Runoff (R) of the Yellow River (YR) basin, source regions (SR), upper reaches (UR), middle reaches (MR) and lower reaches (LR), respectively. **f-j,** Potential evapotranspiration (PET) of the YR basin, SR, UR, MR and LR, respectively. **k-o,** Hydrothermal index (HI) of the YR basin, SR, UR, MR and LR, respectively. **p-t,** Runoff coefficient (RC) of the YR basin, SR, UR, MR and LR, respectively.

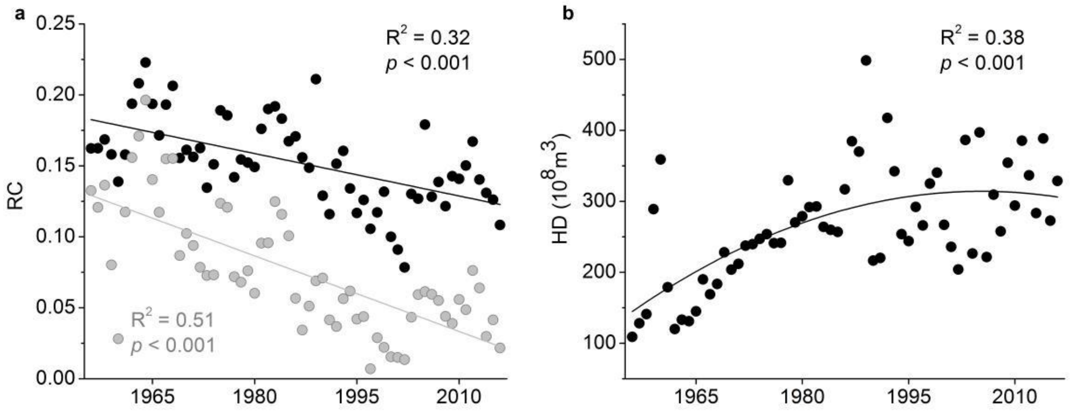

Supplementary Fig. 4 | Values of runoff coefficient (RC) and human disturbance (HD) for the Yellow River in the last six decades. a, Comparison of natural RC (black circle) and actual RC (gray circle). **b,** HD.

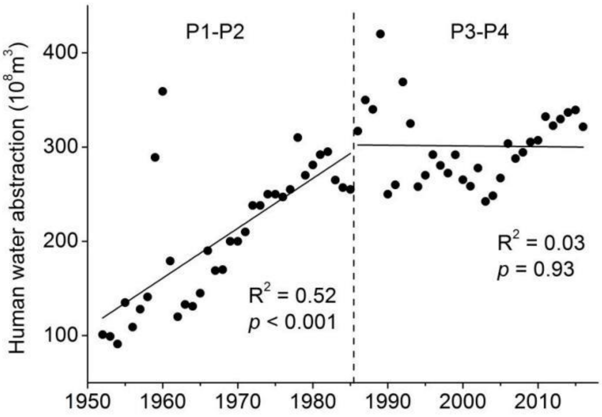

Supplementary Fig. 5 | Values of human water consumption of the Yellow River and its trends in different periods.

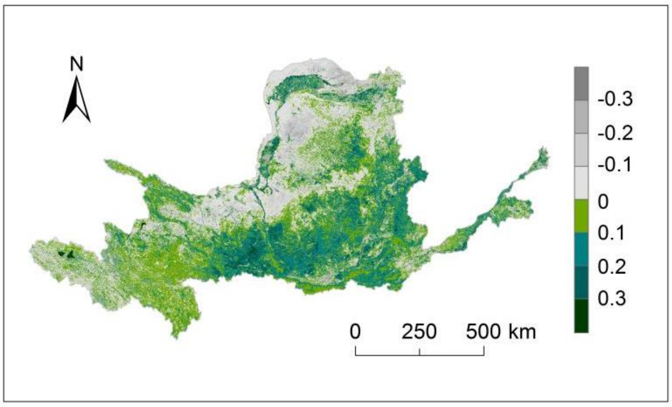

Supplementary Fig. 6 | Difference of the normalized differential vegetation index (NDVI) between 1998 and 2015. More than 61% of the Yellow River basin became greener. New (prepared by YW in ArcMap 10.2, https://www.esri.com/zh-cn/arcgis/products/arcgis-pro/resources).

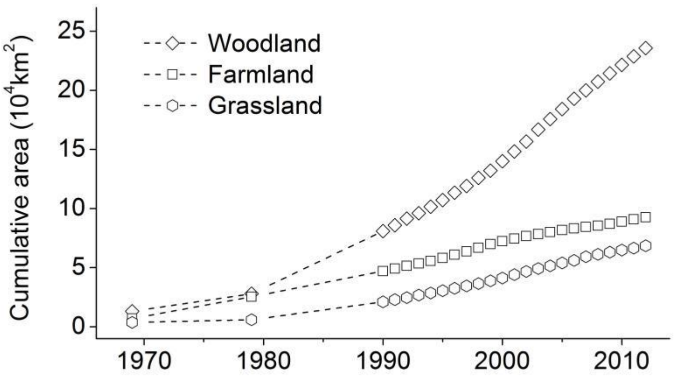

Supplementary Fig. 7 | Cumulative areas of three soil and water conservation measures in the Yellow River basin.

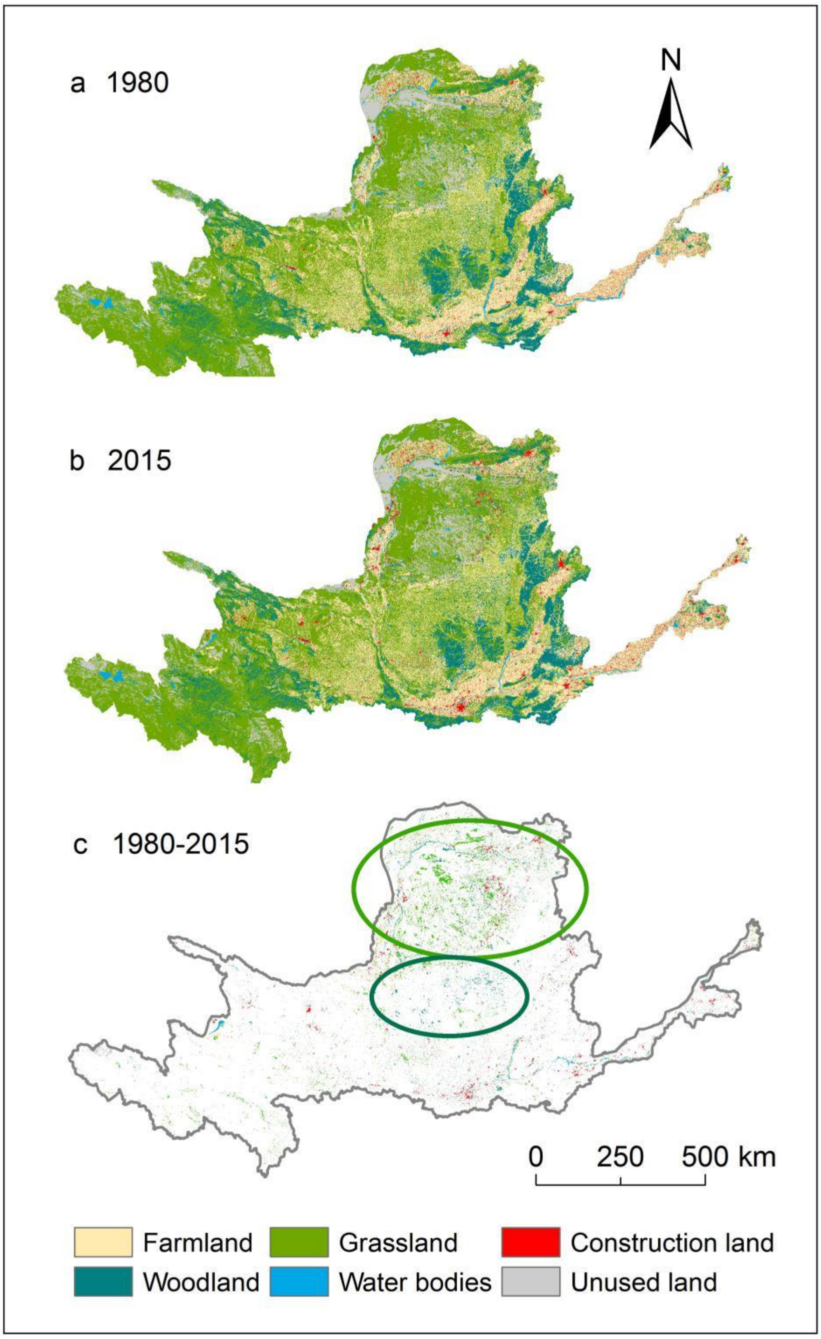

Supplementary Fig. 8 | Land use change of the Yellow River basin from 1980 to 2015. a, Land use in 1980. **b,** Land use in 2015. **c,** Land use change of 2015 compared with 1980. Most of the grassland increase took place in bigger circle, while woodland in smaller one. New (prepared by YW in ArcMap 10.2, https://www.esri.com/zh-cn/arcgis/products/arcgis-pro/resources).

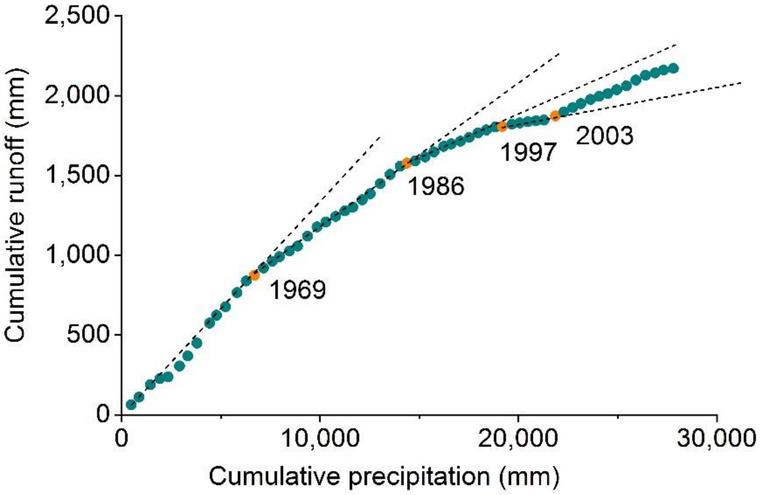

Supplementary Fig. 9 | Relationship between cumulative precipitation and cumulative runoff in the Yellow River basin. Dashed lines show the trends and the orange circles represent turning points in the relationship.

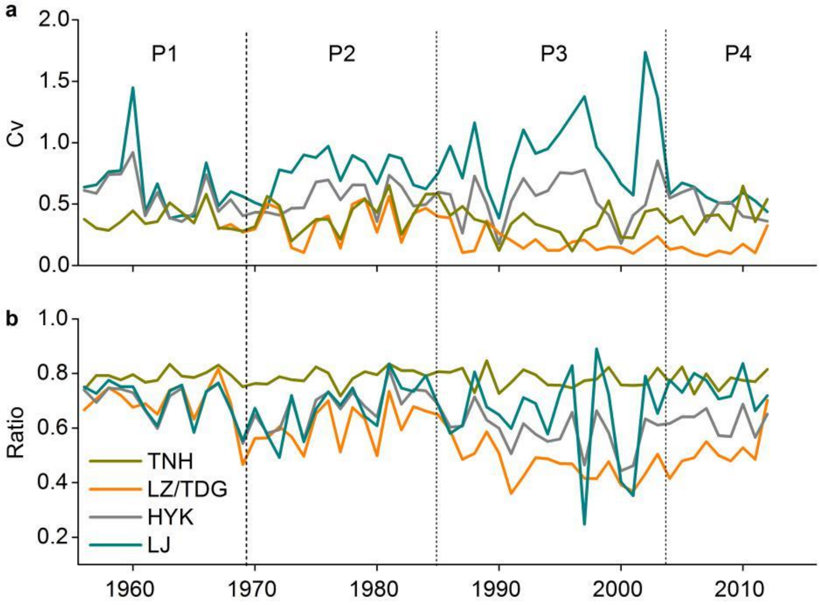

Supplementary Fig. 10 | Regime change of seasonal streamflow relating to reservoirs. a, Coefficient of variation of flood-season streamflow volumes at the main gauging stations. **b,** The ratio of flood-season streamflow to annual streamflow at the main gauging stations. TNH, Tangnaihai; LZ, Lanzhou; TDG, Toudaoguai; HYK, Huayuankou; LJ, Lijin.

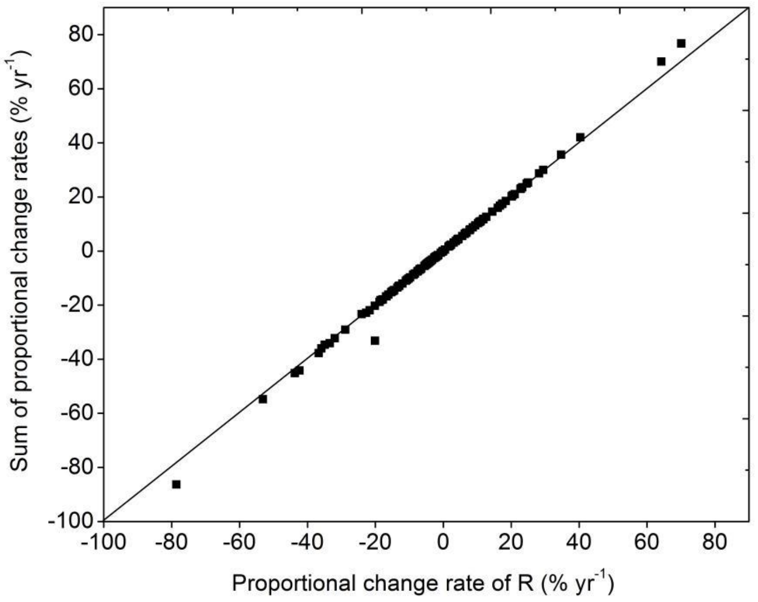

Supplementary Fig. 11 | Comparison of the proportional change rate of runoff (R) with the sum of proportional change rates of potential evapotranspiration (PET), hydrothermal index (HI) and runoff coefficient (RC). In theory these should be equal, but in practice there is a slight deviation due to the calculation of runoff identity factors decomposition. The disagreement is within our acceptable limits.

Supplementary Table 1 | Characteristics of the sub-basins of the Yellow River basin.

	Source regions	Upper reaches	Middle reaches	Lower reaches	Total
Area (10^4 km^2)	12.40	27.30	34.47	3.00	77.17
Number of large tributaries (area > 10^3 km^2)	25	18	30	3	76
Annual runoff (mm)	160.45	2.75	43.15	-266.15	35.60
Annual rainfall (mm)	498.89	327.56	522.89	672.73	455.76
Annual potential evapotranspiration (mm)	827.86	1004.53	998.56	1066.04	975.86
Population (10^4)	65.23	2523.39	7330.75	1391.90	11311.27
Farmland (10^4 km^2)	0.08	4.56	10.16	1.13	15.93
Irrigation area (10^4 km^2)	0.02	1.87	2.50	2.93	7.32

Supplementary Table 2 | Proportional change rates of runoff identity factors of the Yellow River basin (YR), source regions (SR), upper reaches (UR), middle reaches (MR) and lower reaches (LR).

Rate (%yr^{-1})	YR	SR	UR	MR	LR
PET	0.006	0.092	0.005	-0.008	-0.109
HI	-0.088	0.048	-0.021	-0.180	-0.031
RC	-2.229	-0.235	-33.152	-1.766	-1.890
Total	-2.311	-0.095	-33.168	-1.954	-2.031

Note: PET, potential evapotranspiration; HI, hydrothermal index; RC, runoff coefficient. The same below.

Supplementary Table 3 | Proportional change rates of natural runoff identity factors in different periods.

Rate (%yr^{-1})	P1-P4	P1-P2	P2-P3	P3-P4
PET	0.006	-0.128	-0.063	0.148
HI	-0.088	0.072	-0.170	0.245
RC_n	-0.597	0.037	-1.303	-0.629
Total	-0.679	-0.019	-1.537	-0.237

Supplementary Table 4 | Proportional change rates of runoff identity factors between two consecutive years.

Rate (%yr^{-1})	PET	HI	Actual RC		Natural RC	
			RC	Total	RC_n	Total
1956-1957	1.314	-10.154	-4.686	-13.527	0.009	-8.832
1957-1958	-1.159	18.636	6.070	23.546	2.824	20.300
1958-1959	-0.343	-7.860	-25.909	-34.112	-2.813	-11.017
1959-1960	0.928	-7.679	-48.050	-54.802	-7.808	-14.559
1960-1961	-2.137	17.484	61.340	76.688	7.800	23.148
1961-1962	1.864	-18.337	14.054	-2.419	9.716	-6.757
1962-1963	-1.173	7.228	4.613	10.668	2.650	8.704
1963-1964	-4.244	20.239	6.913	22.907	4.162	20.156

1964-1965	6.570	-35.084	-16.649	-45.163	-7.489	-36.003
1965-1966	-0.122	12.762	-8.946	3.694	-5.807	6.833
1966-1967	-4.698	16.175	13.827	25.305	6.024	17.502
1967-1968	2.598	-12.893	0.121	-10.173	2.944	-7.350
1968-1969	2.662	-6.659	-28.287	-32.284	-16.247	-20.244
1969-1970	-1.972	4.069	8.195	10.292	4.245	6.342
1970-1971	0.903	-1.837	-4.290	-5.224	-1.438	-2.373
1971-1972	1.466	-10.552	-8.921	-18.007	2.448	-6.638
1972-1973	-1.436	16.994	-3.810	11.748	-10.066	5.492
1973-1974	0.144	-10.132	0.249	-9.739	5.955	-4.033
1974-1975	-2.708	12.676	25.640	35.608	10.706	20.674
1975-1976	-0.466	-1.617	-1.156	-3.239	0.536	-1.546
1976-1977	1.992	-5.780	-25.344	-29.132	-10.984	-14.772
1977-1978	0.423	4.634	-2.750	2.307	-0.680	4.377
1978-1979	-0.401	-3.165	5.584	2.018	1.257	-2.310
1979-1980	0.037	-6.251	-11.698	-17.912	-0.262	-6.476
1980-1981	-0.194	7.437	22.720	29.962	7.307	14.549
1981-1982	-0.272	-7.430	0.095	-7.607	4.141	-3.562
1982-1983	-2.657	14.399	13.195	24.938	0.894	12.637
1983-1984	0.281	-1.328	-3.715	-4.762	-3.194	-4.241
1984-1985	-0.169	0.238	-6.969	-6.900	-4.417	-4.348
1985-1986	2.207	-18.403	-28.011	-44.207	1.260	-14.936
1986-1987	1.256	5.193	-24.588	-18.139	-8.395	-1.946
1987-1988	-3.288	12.320	19.670	28.703	-2.367	6.665
1988-1989	-1.436	-2.462	14.878	10.979	15.555	11.656
1989-1990	2.739	0.328	1.401	4.467	-16.126	-13.060
1990-1991	0.914	-12.447	-26.242	-37.774	-2.906	-14.438
1991-1992	-2.103	12.404	-5.984	4.318	1.555	11.857
1992-1993	-0.167	-4.928	21.019	15.924	6.947	1.852
1993-1994	2.906	0.421	4.646	7.973	-8.294	-4.967
1994-1995	-0.051	-3.646	-19.166	-22.864	-4.614	-8.311
1995-1996	-2.212	6.448	2.101	6.337	0.304	4.540
1996-1997	3.866	-17.485	-72.722	-86.340	-2.506	-16.124
1997-1998	-1.588	16.993	61.374	76.779	1.326	16.731

1998-1999	0.519	-9.007	-13.429	-21.918	5.841	-2.647
1999-2000	-0.126	0.975	-17.676	-16.827	-13.713	-12.864
2000-2001	0.298	-1.099	-1.375	-2.176	-4.739	-5.540
2001-2002	-0.579	1.065	-5.721	-5.234	-7.398	-6.911
2002-2003	-2.974	20.152	52.853	70.030	24.853	42.030
2003-2004	3.429	-17.364	15.569	1.633	-1.316	-15.252
2004-2005	-0.044	0.345	1.671	1.972	17.061	17.362
2005-2006	0.788	-3.073	-1.505	-3.790	-16.544	-18.828
2006-2007	-2.063	8.999	-3.849	3.087	3.904	10.840
2007-2008	0.155	-5.652	-11.311	-16.808	-6.563	-12.060
2008-2009	0.056	1.374	-5.986	-4.556	8.021	9.451
2009-2010	0.301	0.331	17.829	18.462	-0.661	-0.029
2010-2011	-1.158	5.753	-6.923	-2.329	3.222	7.817
2011-2012	-0.211	-0.930	22.151	21.010	5.320	4.179
2012-2013	3.266	-3.244	-8.802	-8.779	-8.710	-8.688
2013-2014	-1.932	3.704	-36.456	-34.684	-3.471	-1.699
2014-2015	1.248	-10.004	16.441	7.684	-1.877	-10.633
2015-2016	0.592	7.528	-31.505	-23.385	-7.596	0.524

Supplementary Table 5 | Brief introduction to the major reservoirs in the Yellow River.

	Longyangxia	Liujiaxia	Xiaolangdi
Location	Upper reaches	Upper reaches	Middle reaches
Drainage area (10^4 km^2)	13.14	18.18	69.42
Annual streamflow (10^8m^3)	212	277	377.5
First impounding year	1986	1968	1999
Water level (m)	2600	1735	275
Storage capacity (10^8m^3)	247	57	126.5
Available storage capacity (10^8m^3)	193.5	35	51
